# Case report: late adverse reactions in an epilepsy patient on combination therapy with valproate and lamotrigine

**DOI:** 10.1186/s42494-025-00217-3

**Published:** 2025-04-30

**Authors:** Hui Sang, liqiao Zhao, Yanhua Zhang, Xiaolong Wang, Xiaodong Zhang

**Affiliations:** Department of Neurology, Taiyuan City Central Hospital, The Ninth Clinical Medical College of Shanxi Medical University, Taiyuan, 030001 China

**Keywords:** Lamotrigine, Valproate, Adverse reactions

## Abstract

**Background:**

Late adverse reactions associated with the combined therapy of valproate and lamotrigine are infrequently documented within the Chinese population.

**Case presentation:**

This case report describes a 54-year-old female patient who developed adverse reactions following long-term therapy with valproate and lamotrigine, with symptoms emerging five months after the final adjustment of her antiseizure regimen. The patient presented with symptoms of dizziness, ataxia, nystagmus, and postural tremors. Following blood drug concentration monitoring and subsequent minor dosage adjustments to the antiseizure regimen without medication withdrawal, the patient's symptoms were successfully resolved.

**Conclusions:**

This article underscores the importance of vigilance among clinicians regarding the potential for late adverse reactions and advocates for the proactive monitoring of blood drug concentrations.

**Supplementary Information:**

The online version contains supplementary material available at 10.1186/s42494-025-00217-3.

## Background

Epilepsy is a neurological disorder characterized by recurrent seizures, affecting 9.6 million people in China and approximately 40,000 new cases each year [1]. Valproate (VPA) and lamotrigine (LTG), as broad-spectrum antiseizure medications, are often used in combination for epilepsy management in the Chinese population, which can effectively reduce the frequency of epileptic seizures with relatively fewer side effects. Moreover, with moderate pricing and high cost-effectiveness, they have a high combined usage rate among the Chinese population. However, LTG is primarily metabolized in the liver through glucuronic acid conjugation, while VPA, as a hepatic enzyme inhibitor, can slow down the metabolism of LTG, resulting in a prolonged half-life and potentially increasing the incidence of adverse reactions [2]. The majority of reported adverse reactions manifest during the initial phases of drug introduction or titration. We observed a case of an epilepsy patient who may have developed late side effects induced by LTG after being co-administered with VPA.

## Case presentation

A 54-year-old female patient, who has been experiencing generalized seizures for the past 30 years, has been diagnosed with cryptogenic epilepsy at a local medical facility. The patient has previously used valproic acid, phenobarbital, phenytoin, and carbamazepine irregularly (with specific dosages unable to be recalled), and despite this, the control of epileptic seizures has been suboptimal, with one seizure occurring monthly. Over the past year, the patient has gradually transitioned to a combination of sodium valproate (1000 mg per day, taken in two divided doses) and lamotrigine (200 mg per day, taken in two divided doses) in their antiseizure medication regimen. Following this adjustment, the patient has not experienced any further seizures. Five months later, the patient started to develop symptoms of dizziness, unsteady gait, and postural tremors approximately one hour post-medication, which would gradually subside after about six hours. However, the condition has been deteriorating over time, significantly impacting the patient's daily activities.

She has no family history of neurological diseases, denies any history of hypertension, diabetes, brain injury, or drug allergies, and demonstrates normal growth and development. On admission, her vital signs were stable. Neurological examination revealed decreased memory and calculation abilities, vertical down-looking nystagmus, postural tremor, and inaccurate ataxia tests, while other neurological examinations were normal (as depicted in the VideoS1-3). No abnormalities were found in the laboratory blood tests and brain magnetic resonance imaging. The video electroencephalogram revealed diffuse 6–7 Hz slow waves across the entire brain during the interictal period, with sporadic sharp-slow wave complexes in the bilateral frontal and anterior temporal regions, and no epileptiform discharges were observed. We ruled out other diseases associated with cerebellar ataxia.

Considering the close temporal correlation between the emergence of the aforementioned symptoms and the intake of medication, we monitored the blood concentrations of the antiseizure medications. Serum concentrations of LTG and VPA were determined at peak and trough levels. The VPA levels were found to be 98.28μg/mL at peak and 90.45μg/mL at trough, both falling within the therapeutic range of 50 to 100μg/mL. Conversely, the LTG levels were 18.67μg/mL at peak and 14.93μg/mL at trough, exceeding the therapeutic range of 3 to 14 μg/mL in both instances (Table 1).

**Table 1:** Serial lamotrigine and valproate serum levels

Date and time	Lamotrigine serum level (therapeutic range: 3–14 μg/mL^a^)	Valproate serum level (therapeutic range: 50–100μg/mL^a^)
12/01/2023 09:34 (at peak)	18.67	98.28
12/01/2023 17:47 (at trough)	14.93	90.45
19/1/2023 09:30 (at peak, adjusted)	13.54	95.23
19/1/2023 18:00 (at trough, adjusted)	12.19	90.15

^a^Test performed at KingMed diagnostics inc

Attributing the above symptoms to side effects of LTG based on blood concentration results, the daily dosage of LTG was reduced from 200 to 150 mg, while the VPA dosage was maintained at 1000 mg/day. Within a week, the symptoms resolved without any epileptic seizures occurring. Upon re-evaluation of the serum drug concentrations, the VPA levels were found to be 95.23 μg/mL at the peak and 90.15 μg/mL at the trough, and the LTG levels were 13.54 μg/mL at the peak and 12.19 μg/mL at the trough, all of which fall within the therapeutic range (Table 1). At the three-month follow-up, the patient reported no recurrence of the aforementioned symptoms, demonstrated self-sufficiency in daily activities, and remained free from epileptic seizures.

## Discussion

The epilepsy patient experienced central nervous system side effects closely associated with the timing of medication intake five months after the adjustment of antiseizure medications. Upon monitoring of blood drug concentrations, it was hypothesized that the side effects could be associated with LTG, despite the drug being administered at a dosage within the therapeutic range.

After a minor reduction in the LTG dosage, the side effect symptoms gradually subsided, and subsequent blood drug concentration measurements returned to the normal range, thereby validating the aforementioned hypothesis.

LTG acts on voltage-gated sodium channels, indirectly modulating the release of presynaptic neurotransmitters such as aspartate and glutamate, thereby inhibiting the onset of seizures [2]. LTG metabolism primarily involves enzyme-catalyzed reactions in the liver. When co-administered with hepatic enzyme inhibitors such as VPA, it can slow down the metabolism of LTG, increase blood drug concentrations, prolong the half-life, and raise the risk of adverse reactions due to drug accumulation [2]. The blood concentration of LTG rises with the increase in the blood concentration of VPA, showing a significant positive correlation [3]. When LTG is used in combination with VPA, it is recommended to reduce the dosage of LTG, with the target dosage being reduced to 50% of the monotherapy dosage [4].

LTG exhibits significant inter-individual variability in adverse reactions, which often emerge within 6 to 8 weeks of drug initiation, with rash being the most common [5]. Hehe et al. reported that in the Chinese population, the frequent central nervous system side effects of LTG use encompass somnolence, ataxia, headaches, dizziness, nausea, vomiting, memory impairment, and tremors, with the majority of these adverse reactions manifesting within the initial 28 days following LTG initiation [6]. Late side effects are less frequently reported.

Several case reports have delineated the delayed-onset central nervous system adverse reactions associated with the concurrent administration of VPA and LTG. Thome-Souza et al. described four cases of patients who experienced late adverse reactions after the co-administration of two drugs, with the time interval from the last dosage adjustment to the occurrence of adverse reactions varying from 9 months to 2 years. The patients presented with a range of adverse reactions, encompassing common symptoms like ataxia, vertigo, and headaches, alongside rarer manifestations such as tics, ocular motor abnormalities, and dyskinesias. Symptoms were promptly alleviated following a modest reduction in LTG dosage (25–50 mg), without necessitating medication withdrawal or experiencing seizure exacerbation [7]. The symptoms exhibited in this case closely resemble those of the patient detailed within this manuscript, yet they have not been documented among the Chinese population previously. This phenomenon is potentially related to ethnicity and necessitates further exploration into the underlying mechanisms.

Tremors associated with VPA and LTG treatment are predominantly of the postural and intention types (with resting tremors being an uncommon manifestation [3, 8, 9]), as illustrated by this case. While the underlying mechanism remains elusive, it is hypothesized that movement disorders and ocular motor crises may be associated with the modulation of dopamine metabolism through the inhibition of glutamate [7]. Additionally, it is considered that cerebellar pathways may be involved in its pathological mechanism [10]. A study indicates that long-term use of LTG may be associated with a reduction in cerebellar volume [11].

In clinical practice, the combination therapy of LTG and VPA exerts a favorable effect on the control of epileptic seizures, and most physicians closely monitor for adverse reactions during the process of medication initiation or titration. Given the varying physiological and pathological conditions of different patients, as well as the presence of genetic polymorphisms, the pharmacokinetic behavior and optimal therapeutic concentrations of LTG may still exhibit significant inter-individual differences even when administered according to the recommended dosing regimen. Monitoring blood concentration provides valuable information about individual variability in drug metabolism. By regularly checking the blood concentration, we can anticipate potential breakthrough seizures before they occur and make preemptive adjustments to the dosage. It also helps in ensuring that the drug levels remain within the therapeutic range, minimizing the risk of toxicity [3]. Therefore, clinicians administering the aforementioned combination therapy for epilepsy should remain vigilant for potential late adverse effects and are encouraged to conduct blood drug level monitoring.

## Conclusions

Chinese patients undergoing long-term therapy with lamotrigine and valproate require vigilant monitoring for potential late adverse reactions, and proactive blood drug concentration monitoring is encouraged throughout the treatment process. We have demonstrated that timely detection of these adverse events is crucial, and a minor reduction in the dosage of antiseizure medications can lead to symptom alleviation without exacerbating epileptic seizures.

## Supplementary Information

Supplementary Material 1.

Supplementary Material 2.

Supplementary Material 3.

## Data Availability

No datasets were generated or analysed during the current study.

## Abbreviations

LTG: Lamotrigine
VPA: Valproate
